# Role of microRNA-132 in Opioid Addiction through Modification of Neural Stem Cell Differentiation

**DOI:** 10.3390/jpm12111800

**Published:** 2022-11-01

**Authors:** Anne-Marie Fauser, Emily Stidham, Craig Cady, Ashim Gupta

**Affiliations:** 1 Bohlander Stem Cell Research Laboratory, Biology Department, Bradley University, Peoria, IL 61625, USA; 2 Regenerative Orthopaedics, Noida 201301, India; 3 Future Biologics, Lawrenceville, GA 30043, USA; 4 South Texas Orthopaedic Research Institute (STORI Inc.), Laredo, TX 78045, USA

In this editorial, we focused on the article, “MicroRNA-132 in the Adult Dentate Gyrus is Involved in Opioid Addiction Via Modifying the Differentiation of Neural Stem Cells” by Jia and colleagues. This article was intriguing as it investigated the connection between opioid addiction and neuronal differentiation through microRNA (miRNA), i.e., miRNA-132 [1].

Drug addiction can be categorized as a brain disorder in which the expression patterns of genes involved in neural plasticity (the process of stimuli-based changes in neuronal circuits) are altered. Drug addiction has been associated with serious social and public health impacts. In the United States, opioid addiction leads to an enormous health burden (costing billions of dollars) and causes thousands of deaths every year. The most commonly abused opiates include morphine, heroin, and oxycodone [2].

Animal models have shown that adolescent rodents were more likely to develop opioid addiction due to an enhanced feedback mechanism from the µ opioid receptor (MOR) and fewer opioid withdrawal symptoms [3,4]. Some molecular genetic studies suggested that opioid addiction is not only initiated by environmental elements but may also have heritable factors [5]. Hence, it may be postulated that genetic profiles may affect the likelihood of opioid addiction in certain populations. Moreover, drug abuse has been indicated to alter the gene expression processes involved in neural plasticity, memory, learning, and motivational behaviors [6]. In addition, the role of miRNAs to regulate gene expression in neurogenesis has already been thoroughly described in the published literature [7,8].

Neurogenesis is a complicated process involving neural stem cell (NSC) proliferation (self-renewal), differentiation (fate specification), migration, neuronal maturation, and functional neuronal integration. NSCs are progenitor cells that can proliferate and give rise to both neuronal and glial lineages. The whole process of neurogenesis is regulated by the dynamic interplay between transcription factors, miRNAs, and cell-niche signaling [9]. A major portion of NSCs in the adult mammalian brain is located in the ventricular-subventricular zone (V-SVZ) of the lateral ventricles (LVs). Young neurons produced by these basic progenitors grow as far as the olfactory bulb (OB) [10]. Additionally, NSCs are also found in the hippocampus’s subgranular zone (SGZ), where they produce new excitatory neurons for the dentate gyrus (DG) that are critical to learning, memory, and pattern recognition [11]. In the mammalian brain, the hippocampus is crucial for learning and memory development [12]. A visual representation of the processes and site of neurogenesis can be found in Figure 1.

Neuronal plasticity in the hippocampus has been investigated to comprehend the molecular underpinnings of learning and memory. The alterations in neuronal circuits can be functional or structural and manifest through changes in morphology, synaptic connectivity, or genetic expression [13]. Moreover, repeated drug exposure can induce neuronal plasticity, ultimately leading to behavioral changes, including drug sensitization and drug-seeking behavior [14]. According to several studies, the hippocampus is thought to have a role in the emergence and maintenance of addiction [15,16]. Preclinical research, for instance, indicated that early exposure to drugs and alcohol may alter hippocampus function, resulting in increased drug–context connections, aiding in the development of addiction. One particular mechanism studied to understand learning and memory is long-term potentiation (LTP). The retrograde messenger nitric oxide (NO) is thought to induce LTP in the CA1 region of the hippocampus via the activation of soluble guanylyl cyclase (sGC) and, ultimately, cGMP-dependent protein kinase (cGK). Thus, increasing doses of cocaine and nicotine increase LTP in the CA1 area of the hippocampus, whereas neurotoxic amounts of methamphetamine decrease it [17,18,19]. Various studies have underscored the role of the synaptic alterations in the hippocampus by stimulants in the association of pleasure with drug-associated memories and eventual addiction to the drug [20,21]. While stimulants have been shown to enhance LTP in the CA1 region, alcohol and drugs (opiates, cannabinoids) that are central nervous system depressants indicate decreased CA1 LTP. For instance, chronic ethanol exposure impairs hippocampus CA1 LTP, according to research utilizing animal models exposed to moderate to severe alcohol use [22].

Previously, a significant number of studies presented investigations pertaining to the role of miRNAs in many processes of the central nervous system, including neuronal development and differentiation [23], synaptic formation, neuronal plasticity, and the regulation of memory, cognition, and emotion [24,25,26]. MicroRNAs are small (usually 19–22 nucleotide long) non-coding RNA transcripts that regulate gene expression. They were first discovered in *Caenorhabditis elegans* in 1993 [27]. They affect the translation and stability of their mRNA targets by guiding RNA-induced silencing complex (RISC) predominantly to 3′UTR of mRNAs [28]. MiRNAs are particularly abundant in the nervous system. Interestingly, Gu et al. explored the expression pattern of circulating miRNAs in subjects with drug addiction and tested the potential of altered serum miRNAs as noninvasive diagnostic tools for drug abuse. Through microarray analysis, they identified altered levels of miRNA in heroin abusers [29]. Furthermore, Silveira et al. reviewed the existing literature and reported that there are at least 55 miRNAs upregulated and involved in neuronal differentiation, suggesting the involvement in multiple genetic elements during this complex process [30]. A non-comprehensive list can be found in Figure 2.

Subsequently, there is growing interest in the significance of the research to determine the effect of miRNA-132 on the differentiation of neural stem cells (NSCs). miRNA-132 is an important regulator of neural activity, and it has been shown to regulate neurogenesis in adult mouse brains, as well as in human NSCs. For instance, Walgrave et al. using a mouse model, determined that restoring the in vivo expression of miRNA-132 in adult NSCs rescued neurogenic deficits in Alzheimer’s disease [31]. In this study, the researchers investigated whether miRNA-132 can regulate adult hippocampal neurogenesis in healthy and Alzheimer’s brains. Using a distinct Alzheimer’s amyloid mouse model, cultured human neural stem cells, and post-mortem human brain tissue, it was discovered that this RNA molecule is required for the neurogenic process in the adult hippocampus. These findings highlighted the therapeutic potential of miRNA-132 to restore neurogenesis and inhibit the progression of neurodegenerative diseases such as Alzheimer’s disease [31].

The article, entitled “MicroRNA-132 in the Adult Dentate Gyrus is Involved in Opioid Addiction Via Modifying the Differentiation of Neural Stem Cells”, further explored the role of miR-132 in the process of NSCs differentiation. Jia and colleagues designed this study to investigate if miR-132 influences the differentiation of NSC-like cells (Neuro 2A cells) from adults and NSCs from embryonic tissue. The effect of miR-132 in the NSCs of the adult DG on morphine addiction was also assessed in this study using the Sprague Dawley rat morphine self-administration paradigm. In these experiments, overexpression of miRNA-132 was accomplished by transfection using the vectors pCMV-MIR and lentivirus pLenti-CMV-mir-132. Overexpression of miR-132 resulted in enhanced differentiation of N2a cells and NSCs. This enhancement of differentiation was observed both in the in vitro cultured NSCs, as well as in vivo NSCs, in the adult DG. The findings of this study strongly suggested that the miRNA-132 overexpression in the NSCs of adult DG leads to enhanced consolidation and impaired elimination of addiction memory [1].

The primary focus of this article was the role of miRNAs on neurogenesis and the formation of opioid addiction. The initial motivation for the current research might have been previously observed upregulated expression of miRNA-132 with morphine exposure [32]. The issue under study was well described with the assistance of a comprehensive literature review. The problem statement was efficiently constructed to specifically state the hypothesis to be tested. The study design was planned according to the proposed hypothesis, and the results were explained well, with sufficient figures and data charts. However, the study’s scope was limited to testing the effect of miR-132 expression only. In our opinion, it may be worthwhile to include other miRNAs along with miR-132 in this study design to determine the most critical pathway(s) in the complex process of neural stem cell differentiation. Further research in this area may also find significance in the clinical research for the identification of novel molecular therapeutic targets to treat neurodegenerative disorders.

Interestingly, Jia and colleagues recently carried out another study, in which it was noted that in vitro morphine exposure (for 24 h) promotes the differentiation of N2a cells that express the μ-opioid receptor via the upregulation of miR-132 expression. They also observed that in vivo morphine dependence was clearly associated with increased miR-132 expression and neuronal structural plasticity in the DG neurons of rats [33]. These findings again demonstrated a specific link between miR-132 expression and changes in neuronal structure and function upon opioid exposure.

In conclusion, neuronal differentiation and drug-seeking behaviors were increased in miR-132-overexpressed mice when exposed to opioids. This overexpression increased the differentiation of NSCs both in vitro and in vivo. Therefore, it can be concluded that miR-132 is involved in opioid addiction, probably by promoting the differentiation of NSCs in the adult DG. Moreover, recent groundbreaking advances in the treatment of neurodegenerative disorders using small RNA-based therapeutics may pave the way for more direct miR-132-based therapeutic or diagnostic approaches. Future research in the field of addiction biology should continue to investigate miRNAs with a greater focus on the mechanism(s) of how miRNA expression changes over time in response to stimulants.

## Figures and Tables

**Figure 1:**
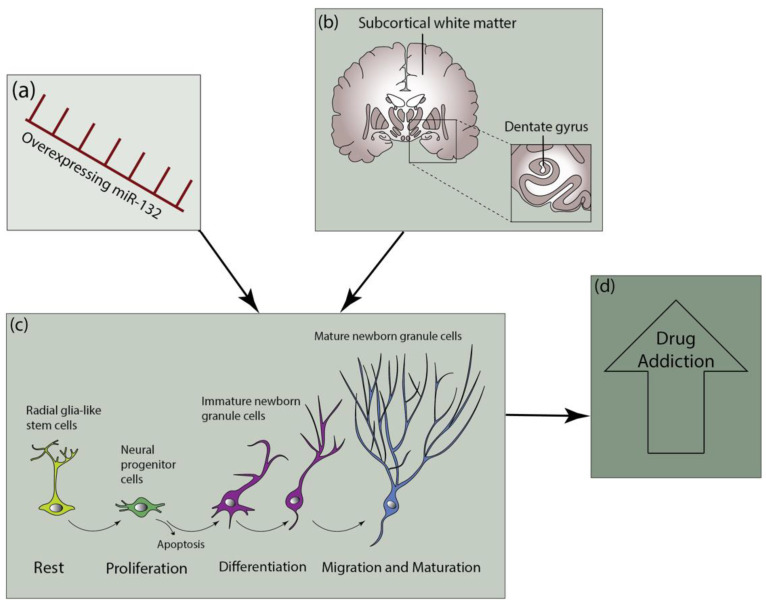
Upregulation of miR-132 (**a**) in the NSCs of the adult DG (**b**) leads to the differentiation, extended dendritic processes, and enhanced morphological development of dendrites (**c**), impairing the elimination of addiction memory consolidating opioid addiction (**d**).

**Figure 2:**
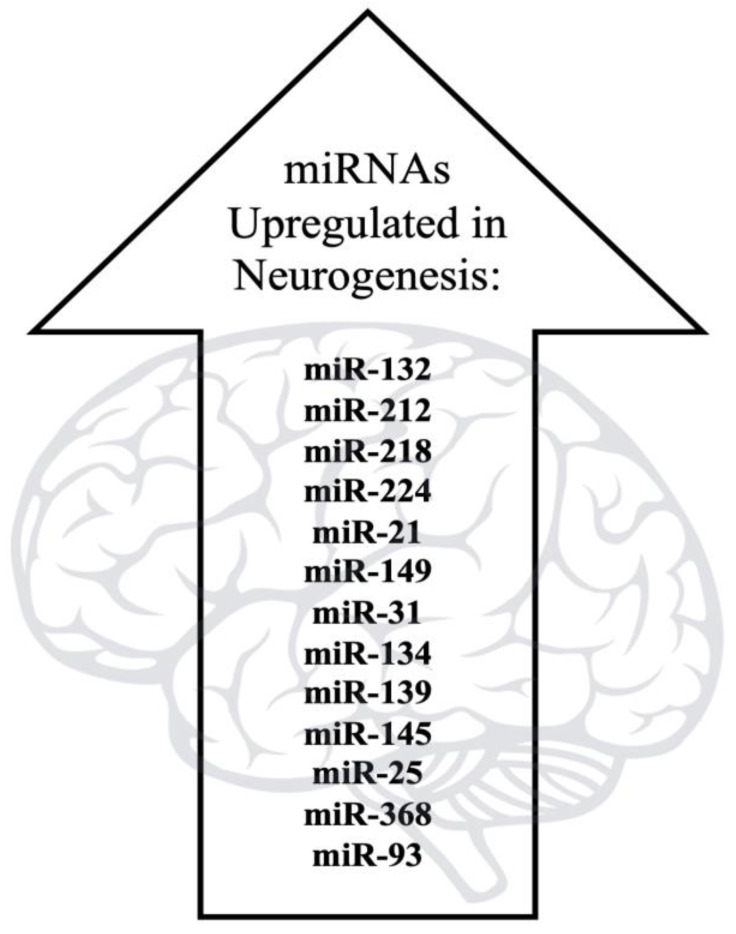
Image documents the various miRNAs that are upregulated during neurogenesis [30].

## Data Availability

The data are contained within the manuscript.
